# Silencing of microRNA-210 inhibits the progression of liver cancer and hepatitis B virus-associated liver cancer via targeting EGR3

**DOI:** 10.1186/s12881-020-0974-9

**Published:** 2020-03-06

**Authors:** Xiaojie Li, Mei Yuan, Lu Song, Yan Wang

**Affiliations:** 1 The seventh Inpatient Area, Qingdao Sixth People’s Hospital, No. 9, Fushun Road, Shibei District, Qingdao City, 266033 Shandong Province China; 2 Department of Inspection, Qingdao Sixth People’s Hospital, No. 9, Fushun Road, Shibei District, Qingdao City, 266033 Shandong Province China; 3 Chronic Disease Management Center, Qingdao Sixth People’s Hospital, No. 9, Fushun Road, Shibei District, Qingdao, 266033 Shandong Province China

**Keywords:** miRNA-210, EGR3, Hepatitis B virus, Liver cancer, Cirrhosis

## Abstract

**Background:**

This study was aimed to investigate the regulatory role of microRNA-210 (miRNA-210) on the progression of liver cancer and Hepatitis B virus (HBV)-associated liver cancer.

**Methods:**

The expression of miRNA-210 was detected in liver tissues of HBV-associated cirrhosis and liver cancer, and in HepG2 and HepG2.2.15 cells by qRT-PCR. MiRNA-210 was silenced in HepG2 and HepG2.2.15 cells by the transfection of miRNA-210 inhibitor. The cell viability and apoptosis was detected by MTT assay and Annexin V-fluorescein isothiocyanate/propidium iodide staining, respectively. The protein expression of EGR3 was detected by Western blot. The regulatory relationship between EGR3 and miRNA-210 was predicted by TargetScan and identified by Dual luciferase reporter gene assay.

**Results:**

MiRNA-210 was overexpressed in the liver tissues of HBV-associated cirrhosis and liver cancer, and in HepG2 and HepG2.2.15 cells (*P* < 0.05). Silencing of miRNA-210 inhibited the viability and promoted the apoptosis of HepG2 and HepG2.2.15 cells (*P* < 0.05). EGR3 was a target of miRNA-210, which was down-regulated in the liver tissues of HBV-associated cirrhosis and liver cancer, and in HepG2 and HepG2.2.15 cells (*P* < 0.05). Silencing of miRNA-210 increased the mRNA and protein expression of EGR3 (*P* < 0.05). Silencing of EGR3 reversed the anti-tumor effect of miRNA-210 inhibitor on HepG2 and HepG2.2.15 cells (*P* < 0.05).

**Conclusions:**

Silencing of miRNA-210 inhibits the progression of liver cancer and HBV-associated liver cancer via up-regulating EGR3.

## Background

Liver cancer, also known as hepatocellular carcinoma (HCC) is a common malignant tumor that associated with high mortality worldwide [1]. Hepatitis B virus (HBV) infection is one of the major causes of liver cancer [2]. In China, about 93 million people are HBV carriers, and about 20 million of them have chronic HBV infection [3]. HBV infection results in the damage on liver tissues and leads to cirrhosis [4]. Cirrhosis caused by HBV infection will further develop into cancer, and then contributes to the poor prognosis [5]. A 10-year follow-up study based on 0.5 million patients with HBV infection in China showed that about 36,000 patients have died due to liver failure, cirrhosis or liver cancer [6].

MicroRNAs (miRNAs) are a class of small noncoding, single-stranded RNAs consisting of 18–25 nucleotides. MiRNAs play important roles in the regulation of diverse cellular biological processes, such as cell differentiation, apoptosis, proliferation, and tumorigenesis [7–9]. Tan et al. [10] showed that the serum miRNA-122-5p, −199a-5p, − 486-5p, −193b-5p, − 206, − 192-5p, − 141-3p and -26a-5p are differentially expressed between cirrhosis and HVB-HCC. Xie et al. [11] proved that serum miRNA-101 level is a candidate biomarker to differentiate HBV-HCC and HBV-cirrhosis. Wang et al. [12] demonstrated that the inhibition of miRNA-15a suppresses the proliferation of HBV-infected HepG2 cells [12]. MiRNA-210 is a hypoxia-regulated-miRNA that participates in the tumorigenesis and progression of HCC [13–15]. However, the specific regulatory effect and mechanism of miRNA-210 on HBV-associated liver cancer remain unclear.

In this study, the expression of miRNA-210 was detected in liver tissues of HBV-associated cirrhosis and liver cancer, and in HepG2 and HepG2.2.15 cells. The effects of miRNA-210 silencing on the cell viability and apoptosis were then analyzed. In addition, the regulatory relationship between EGR3 and miRNA-210 in HepG2 and HepG2.2.15 cells was identified. Our findings may provide a potential therapeutic target for HBV-associated liver cancer.

## Methods

### Participants and liver tissue samples

A total of 25 patients with HBV-associated liver cancer (Liver cancer group, *N* = 25, 13 males and 12 females, 49.3 ± 1.1 years old) and 25 patients with HBV-associated cirrhosis (Cirrhosis group, *N* = 25, 12 males and 13 females, 48.6 ± 1.3 years old) were screened from our hospital between September 2015 and August 2018. A total of 25 healthy participants were enrolled as the Control group (Control group, N = 25, 14 males and 11 females, 48.8 ± 1.2 years old). All participants were diagnosed as HBV-associated cirrhosis or liver cancer at the first time, and no other diseases and tumor metastasis were observed. No significant differences were observed on the age and gender among these three groups. Liver tissues were collected from participants undergoing cancer resection or outpatient liver biopsy. This study was approved by the ethics committee of our hospital, and informed consents were obtained from all participants.

### Cell culture

Human normal liver cell line HL-7702 cells, liver cancer cell line HepG2 cells, and HBV-associated liver cancer cell line HepG2.2.15 cells were purchased from the Institute of Basic Medical Sciences, Chinese Academy of Medical Sciences (Beijing, China). HL-7702 and HepG2 cells were cultured in RPMI1640 medium containing 10% fetal bovine serum (FBS), and HepG2.2.15 cells were cultured in RPMI1640 medium containing 10% FBS and 380 μg/mL G418 (Sigma, Dorset, UK). All cells were maintained in a 5% CO_2_ incubator at 37 °C and 95% humidity.

### Cell transfection

MiRNA-210 inhibitor, miRNA-210 inhibitor negative control (miRNA-210-NC), siRNA1-EGR3 (si1-EGR3), siRNA2-EGR3 (si2-EGR3), and siRNA negative control (si-NC) were purchased from Shanghai GenePharma Co., Ltd. (Shanghai, China). HepG2 and HepG2.2.15 cells were digested with 0.25% trypsin and seeded into 24-well plate at a density of 1.3 × 10^5^ cells/well. When reaching 90% confluence, cells were transfected with the above agents using Lipfectamine 2000 (Thermo Fisher Scientific, Waltham, MA, USA). Cells without transfection were considered as the Blank group. After 48 h of transfection, cells were used for further assays.

### MTT assay

After the transfection, cells were cultured for 7 days and collected every day. The collected cells were seeded into 96-well plates at a density of 1 × 10^4^ cells/well, and then incubated with MTT solution (20 μL, 5 mg/mL) for 4 h at 37 °C. Dimethyl sulfoxide (DMSO, 150 μL) was used to dissolve the MTT formazan crystals. The absorbance at 590 nm (A_590_) was measured by a microplate reader (Thermo Fisher Scientific).

### Annexin V-fluorescein isothiocyanate (FITC)/propidium iodide (PI) staining

After the transfection, cells were cultured for 24 h, and seeded into 24-well plates at a density of 1 × 10^5^ cells/well. After stained with Annexin V- FITC and PI for 15 min under darkness, the apoptosis rate was detected by a Flow Cytometry (BD, San Jose, CA, USA).

### Quantitative real-time PCR (qRT-PCR)

Total RNA was extracted from specific tissues and cells using TRIZOL reagent (Thermo Fisher Scientific). RNA was reverse transcribed into cDNA on a Gene Amp PCR System 9700 (Applied Biosystems, Foster City, CA) using a RevertAidTM H Minus First Strand cDNA Synthesis Kit (Thermo Fisher Scientific, Waltham, MA, USA). qRT-PCR was performed on a Rotor-Gene 3000 Real-time PCR instrument (Corbett Research, Sydney, Australia). The PCR program of miRNA-210 was 95 °C for 10 min, 40 cycles at 95 °C for 15 s and 60 °C for 60 s. The PCR program of EGR3 was 95 °C for 10 min, 35 cycles at 94 °C for 30 s, 53 °C for 45 s and 72 °C for 45 s. U6 was used as an internal reference for miRNA-210, and GAPDH was used as an internal reference for EGR3. The primers sequences were shown as follows: miRNA-210-F: 5′-GTGCAGGGTCCGAGGT-3′, miRNA-210-R: 5′-TATCTGTGCGTGTGACAGCGGCT-3′; U6-F: 5′-CTCGCTTCGGCAGCAC-3′, U6-R: 5′-AACGCTTCACGAATTTGCG-3′; EGR3-F: 5′-TACAATCAGATGGCTACAGAGAAT-3′, EGR3-R: 5′-TTCCCAAGTAGGTCACGGTC-3′; GAPDH-F: 5′-TCGGAGTCAACGGATTTGGTC-3′, GAPDH-R: 5′-GCCATGGGTGGAATCATATTGG-3′. Data was calculated in accordance with the 2^-∆∆Ct^ method.

### Western blot

Total proteins were extracted from specific tissues and cells using RIPA lysis buffer, and then quantified using a Bradford protein assay kit (Beyotime, Shanghai, China). The proteins were separated by sodium dodecyl sulfate polyacrylamide gel electrophoresis (SDS-PAGE), and transferred to polyvinylidene fluoride (PVDF) membrane. After blocked with 5% skim milk for 2 h, the membrane was incubated with Mouse anti-human EGR3 monoclonal antibody (1:1000, Abcam, UK) for 12 h at 4 °C. The membrane was then washed with TBST for 3 times, and subsequently incubated with horseradish peroxidase-labeled goat anti-mouse IgG (l:2000, Zhongshan Jinqiao, Beijing, China) for 1 h at 25 °C. The protein bands were visualized using an ECL kit, and the gray value was quantified by a gel imaging analysis system. GAPDH was used as an internal reference for EGR3.

### TargetScan prediction

The targets of miRNA-210 were predicted using TargetScan 7.1 (http://www.targetscan.org/vert_71/). A total of 4046 transcripts containing 5853 sites were predicted. A target gene EGR3 (ENST00000317216.2) was selected due to its anti-tumor role on liver cancer [16].

### Dual luciferase reporter gene (DLR) assay

The regulatory relationship between EGR3 and miRNA-210 was identified by DLR assay. The sequences of EGR3 wild type (wild) and EGR3 mutation (mutation) were synthesized according to the predicted binding site. The fragments were then inserted to the luciferase reporter vector pGL3-promoter (GenePharma). HepG2 cells were co-transfected with the plasmids carrying wild/mutation and miRNA-210 mimic/miRNA-210 mimic negative control (NC) (GenePharma), and grouped as mimic + wild, mimic + mutation, NC + wild, and NC + mutation group. The fluorescence was detected by a Microplate Reader (Thermo Fisher Scientific). The relative fluorescence unit was calculated as the ratio of Fireny Luciferase and Renilla Luciferase.

### Statistical analysis

All experiments were performed in triplicate. Statistical analysis was performed by SPSS version 17.0 (SPSS Inc., Chicago, IL). Data were expressed as mean ± standard deviation (SD). Differences among multi-groups were analyzed by one-way ANOVA followed by Tukey’s test. A *P*-value of less than 0.05 was considered significantly different.

## Result

### MiRNA-210 was overexpressed in HBV-associated cirrhosis and liver cancer

**Fig. 1:**
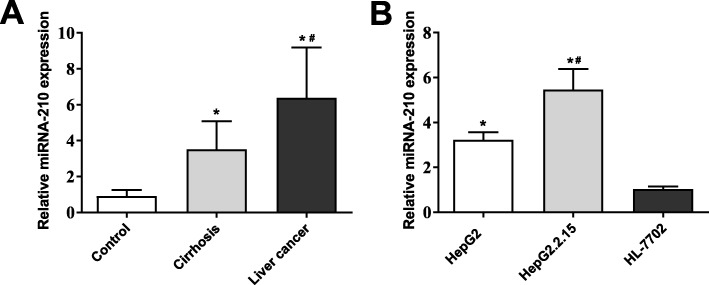
MiRNA-210 was overexpressed in HBV-associated cirrhosis and liver cancer. **a** The expression of miRNA-210 was significantly increased in liver tissues of the Cirrhosis and Liver cancer group. * *P* < 0.05 compared with the Control group. # *P* < 0.05 compared with the Cirrhosis group. **b** The expression of miRNA-210 expression was significantly increased in HepG2 and HepG2.2.15 cells. * *P* < 0.05 compared with HL-7702 cells. # *P* < 0.05 compared with HepG2 cells. The experiment was performed in triplicate, and data were expressed as mean ± standard deviation (SD)

The expression of miRNA-210 was determined by qRT-PCR. The expression of miRNA-210 was significantly higher in liver tissues of the Cirrhosis group than that in tissues of the Control group (*P* < 0.05). The liver tissues in the Liver cancer group exhibited significantly higher expression of miRNA-210 compared with tissues in the Cirrhosis group (*P* < 0.05) (Fig. 1 a). In addition, the expression of miRNA-210 was significantly higher in HepG2 cells than that in HL-7702 cells (*P* < 0.05), and was significantly higher in HepG2.2.15 cells than that in HepG2 cells (*P* < 0.05) (Fig. 1b).

### The transfection of miRNA-210 inhibitor inhibited the expression of miRNA-210 in HepG2 and HepG2.2.15 cells

**Fig. 2:**
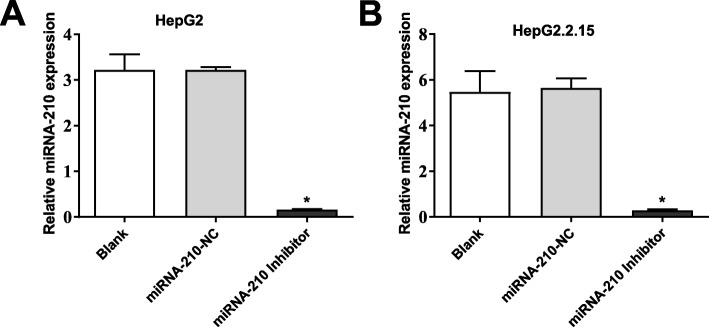
The transfection of miRNA-210 inhibitor inhibited the expression of miRNA-210 in HepG2 **a** and HepG2.2.15 **b** cells. * *P* < 0.01 compared with the Blank group. The experiment was performed in triplicate, and data were expressed as mean ± standard deviation (SD)

MiRNA-210 was silenced in HepG2 and HepG2.2.15 cells by the transfection of miRNA-210 inhibitor. As shown in Fig. 2 a and b, the expression of miRNA-210 was significantly decreased in HepG2 and HepG2.2.15 cells of the miRNA-210 inhibitor group compared with cells of the Blank group (*P* < 0.01). The expression of miRNA-210 in HepG2 and HepG2.2.15 cells was not significantly influenced by the transfection of miRNA-210-NC.

### Silencing of miRNA-210 inhibited the viability of HepG2 and HepG2.2.15 cells

**Fig. 3:**
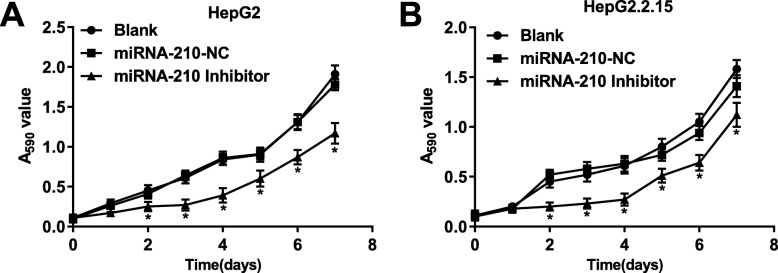
Silencing of miRNA-210 inhibited the viability of HepG2 **a** and HepG2.2.15 **b** cells. * *P* < 0.05 compared with the Blank group. The experiment was performed in triplicate, and data were expressed as mean ± standard deviation (SD)

The cell viability (A_590_ value) was detected by MTT assay. The viability of HepG2 and HepG2.2.15 cells in the miRNA-210 inhibitor group was significantly decreased compared with cells in the Blank group (*P* < 0.05). The viability of HepG2 and HepG2.2.15 cells was not significantly influenced by the transfection of miRNA-210-NC (Fig. 3 a and b).

### Silencing of miRNA-210 promoted the apoptosis of HepG2 and HepG2.2.15 cells

**Fig. 4:**
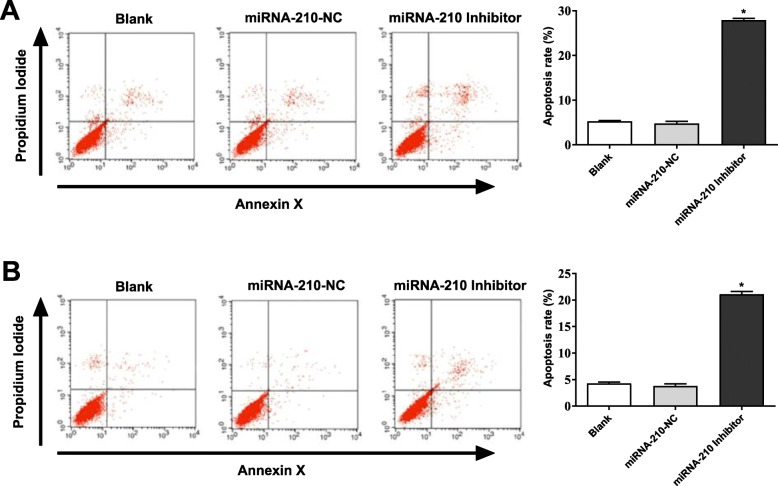
Silencing of miRNA-210 promoted the apoptosis of HepG2 **a** and HepG2.2.15 **b** cells. * *P* < 0.05 compared with the Blank group. The experiment was performed in triplicate, and data were expressed as mean ± standard deviation (SD)

The cell apoptosis was detected by Annexin V-FITC/PI staining. Compared with cells in the Blank group, HepG2 and HepG2.2.15 cells in the miRNA-210 inhibitor group exhibited significantly increased apoptosis rate (*P* < 0.05). The apoptosis of HepG2 and HepG2.2.15 cells was not significantly influenced by the transfection of miRNA-210-NC (Fig. 4 a and b)

### EGR3 was a target of miRNA-210

**Fig. 5:**
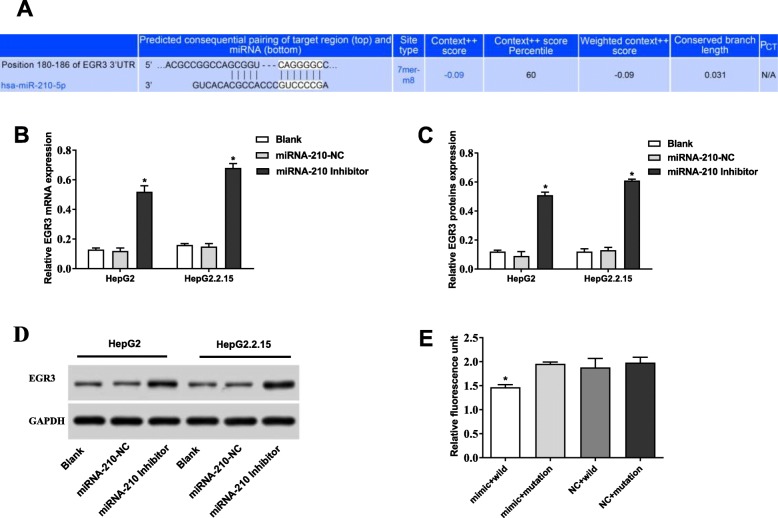
EGR3 was a target of miRNA-210. **a** A binding site at 3′-UTR of EGR3 was predicted on miRNA-210 by Target Scan. **b-d** Silencing of miRNA-210 increased the mRNA and protein expression of EGR3. * *P* < 0.05 compared with the Blank group. **e** The relative fluorescence unit was significantly decreased in the mimic + wild group. * *P* < 0.05 compared with the mimic + mutation group. All experiments were performed in triplicate, and data were expressed as mean ± standard deviation (SD)

A binding site at 3′-UTR of EGR3 was predicted on miRNA-210 by Target Scan (Fig. 5 a). The transfection of miRNA-210 inhibitor significantly increased the expression of EGR3 in HepG2 and HepG2.2.15 cells at both the mRNA and protein level (*P* < 0.05). The expression of EGR3 in HepG2 and HepG2.2.15 cells was not significantly influenced by the transfection of miRNA-210-NC (Fig. 5 b-d). In addition, DLR assay showed that the relative fluorescence unit was significantly decreased in the mimic + wild group compared with that in the other three groups (mimic + mutation, NC + wild, and NC + mutation) (*P* < 0.05) (Fig. 5 e). All these results illustrated that miRNA-210 negatively regulate EGR3 by binding to the 3′-UTR of EGR3.

### EGR3 ***was down-regulated in HBV-associated cirrhosis and liver cancer***

**Fig. 6:**
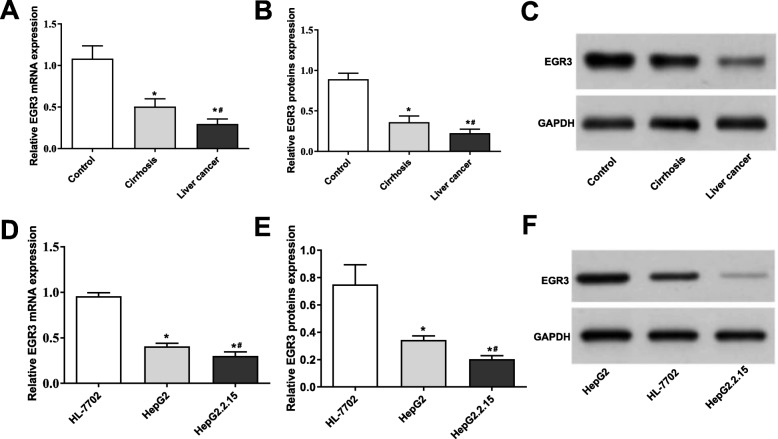
The expression of EGR3 was decreased in HBV-associated cirrhosis and liver cancer. **a-c** The mRNA and protein expression of EGR3 was significantly decreased in liver tissues of the Cirrhosis and Liver cancer group. * *P* < 0.05 compared with the Control group. # *P* < 0.05 compared with the Cirrhosis group. **d-f** The mRNA and protein expression of EGR3 was significantly decreased in HepG2 and HepG2.2.15 cells. * *P* < 0.05 compared with HL-7702 cells. # *P* < 0.05 compared with HepG2 cells. All experiments were performed in triplicate, and data were expressed as mean ± standard deviation (SD)

As shown in Fig. 6 a-c, the mRNA and protein expression of EGR3 were significantly lower in liver tissues of the Cirrhosis group than in tissues of the Control group (*P* < 0.05), and were significantly lower in liver tissues of the Liver cancer group than in tissues of the Cirrhosis group (*P* < 0.05). In addition, the mRNA and protein expression of EGR3 were significantly lower in HepG2 cells than those in HL-7702 cells (*P* < 0.05), and were significantly lower in HepG2.2.15 cells than those in HepG2 cells (*P* < 0.05) (Fig. 6 d-f).

### Silencing of EGR3 ***reversed the anti-tumor effect of miRNA-210 inhibitor on HepG2 and HepG2.2.15 cells***

**Fig. 7:**
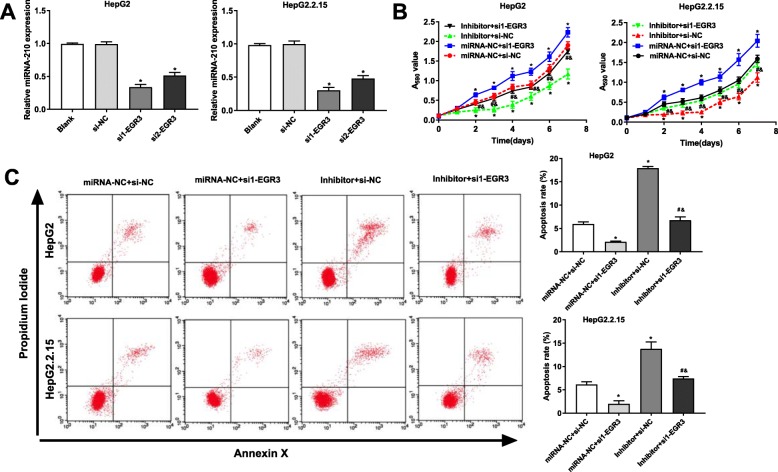
Silencing of EGR3 reversed the effect of miRNA-210 inhibitor on HepG2 and HepG2.2.15 cells. **a** The expression of miRNA-210 was significantly decreased by the transfection of si1- or si2-EGR3. * *P* < 0.05 compared with the Blank group. **b** Silencing of EGR3 reversed the inhibiting effect of miRNA-210 inhibitor on the viability of HepG2 and HepG2.2.15 cells. **c** Silencing of EGR3 reversed the promoting effect of miRNA-210 inhibitor on the apoptosis of HepG2 and HepG2.2.15 cells. * *P* < 0.05 compared with the miRNA + si-NC group. # *P* < 0.05 compared with miRNA-NC + si1-EGR3. & *P* < 0.05 compared with inhibitor + si1-NC. All experiments were performed in triplicate, and data were expressed as mean ± standard deviation (SD)

In order to identify the regulatory relationship between EGR3 and miRNA-210, EGR3 was silenced in HepG2 and HepG2.2.15 cells. qRT-PCR showed that the mRNA expression of EGR3 was significantly decreased by the transfection of si1- or si2-EGR3 in HepG2 and HepG2.2.15 cells (*P* < 0.05) (Fig. 7 a). si1-EGR3 with relatively high silence efficiency was used for further assays. MTT assay showed that the viability of HepG2 and HepG2.2.15 cells was significantly increased in the miRNA-NC + si1-EGR3 group, and was significantly decreased in the inhibitor + si-NC group compared with that in the miRNA-NC + si-NC group (*P* < 0.05) (Fig. 7 b). On the contrary, the transfection of si1-EGR3 significantly decreased the apoptosis rate of HepG2 and HepG2.2.15 cells, and the transfection of miRNA-210 inhibitor significantly increased the apoptosis rate (*P* < 0.05) (Fig. 7 c). Note worthily, the transfection of si1-EGR3 significantly reversed the anti-tumor effect of miRNA-210 inhibitor on HepG2 and HepG2.2.15 cells (*P* < 0.05) (Fig. 7 b and c).

## Discussion

Liver cancer is the third leading cause of cancer death worldwide [17]. Because the cirrhosis is the main cause of liver cancer, the discovery of novel therapeutic target against cirrhosis-induced liver cancer is urgently needed. In this study, we found that miRNA-210 was overexpressed in the liver tissues of HBV-associated cirrhosis and liver cancer, and in HepG2 and HepG2.2.15 cells. Silencing of miRNA-210 inhibited the viability and promoted the apoptosis of HepG2 and HepG2.2.15 cells by targeting EGR3.

Accumulating researches have proved that miRNA-210 plays an important regulatory role in cancer. Eminaga et al. [18] showed that the expression of miRNA-210 is significantly elevated in metastatic prostate cancer. Ke et al. [19] proved that overexpression of miRNA-210 is associated with the development and progression of upper tract urothelial carcinoma. Greither et al. [20] demonstrated that miRNA-210 is correlated with the poor survival of patients with soft-tissue sarcoma. In addition, miRNA-210 is also involved in the occurrence and development of liver cancer. Zhan et al. [21] revealed that the increased serum level of miRNA-210 is a predictive biomarker for the treatment response of transarterial chemoembolization, and for the overall survival of patients with HCC. Yang et al. [13] showed that miRNA-210 is up-regulated in HBV-related HCC, and high expression of miRNA-210 is significantly correlated with the poor prognosis and microvascular density. In this study, the expression of miRNA-210 was significantly increased in the liver tissues of HBV-associated cirrhosis and liver cancer. This result indicates that the overexpression of miRNA-210 is closely correlated with the infection of HBV. This correlation was further identified by that the expression of miRNA-210 was significantly higher in HepG2.2.15 cells than that in HepG2 cells. In addition, silencing of miRNA-210 inhibited the viability and promoted the apoptosis of HepG2 and HepG2.2.15 cells. These findings indicate that miRNA-210 may act as a tumor promoter in liver cancer and HBV-associated liver cancer. We speculated that silencing of miRNA-210 may be a potential therapeutic strategy for HBV-associated liver cancer.

EGR3, an important member of the EGR family, is known as a suppressor of tumor initiation and progression. Sharma et al. [22] indicated that the expression of EGR3 is decreased in tumor tissues of head and neck cancer (HNC), and overexpression of EGR3 significantly inhibits the colony forming ability of HNC cells in vitro. Liao et al. [23] showed that the expression of EGR3 is significantly down-regulated in gastric cancer tissues, and the low expression is positively correlated with the poor prognosis. Note worthily, the abnormal expression of EGR3 is also observed in liver cancer. Zhang et al. [16] showed that the expression of EGR3 is frequently down-regulated in HCC tissues and cell lines, and the ectopic expression of EGR3 inhibits the proliferation and induce the apoptosis of HCC cells in vitro. In this study, EGR3 was identified as a target of miRNA-210. The expression of EGR3 was down-regulated in the liver tissues of HBV-associated cirrhosis and liver cancer, which was contrary to miRNA-210. In addition, silencing of miRNA-210 increased the mRNA and protein expression of EGR3 in HepG2 and HepG2.2.15 cells, indicating that EGR3 was negatively regulated by miRNA-210. Note worthily, silencing of EGR3 reversed the anti-tumor effect of miRNA-210 inhibitor on HepG2 and HepG2.2.15 cells. We speculated that silencing of miRNA-210 may inhibit the viability and promote the apoptosis of HepG2 and HepG2.2.15 cells through up-regulating EGR3. Our findings provide a theoretical basis for the targeted therapy of HBV-associated liver cancer.

## Conclusions

In conclusion, overexpression of miRNA-210 was closely correlated with HBV-associated cirrhosis and liver cancer. Silencing of miRNA-210 inhibited the viability and promoted the apoptosis of HepG2 and HepG2.2.15 cells through up-regulating EGR3. MiRNA-210 and its target EGR3 may be potential therapeutic targets for HBV-associated liver cancer.

## Data Availability

The genes analyzed in the present study are available at https://www.ncbi.nlm.nih.gov/search/ with accession number of ID 406992 (microRNA-210, ENSG00000199038, http://asia.ensembl.org/Homo_sapiens/Gene/Summary?db=core;g=ENSG00000199038;r=11:568089-568198;t=ENST00000362168), ID1960 (Early growth response 3, ENSG00000179388, http://asia.ensembl.org/Homo_sapiens/Gene/Summary?db=core;g=ENSG00000179388;r=8:22687659-22693480), and ID2597 (Glyceraldehyde-3-phosphate dehydrogenase, ENSG00000111640, http://asia.ensembl.org/Homo_sapiens/Gene/Summary?db=core;g=ENSG00000111640;r=12:6534512-6538374). The other data analyzed in the current study are available from the corresponding author on reasonable request.

## Abbreviations

HBN: Hepatitis B virus
HCC: Hepatocellular carcinoma
miRNAs: MicroRNAs
